# The Role of Hepcidin in Regulating Iron Homeostasis in Selected Diseases

**DOI:** 10.34763/devperiodmed.20192302.137141

**Published:** 2019-07-08

**Authors:** Aleksandra Radosz, Anna Obuchowicz

**Affiliations:** 1 Chair and Department of Pediatrics in Bytom, The School of Health Sciences, Medical University of Silesia, Katowice, Poland

**Keywords:** iron metabolism, hepcidin, infection diseases, metabolic diseases, chronic diseases, gospodarka żelazem, hepcydyna, choroby infekcyjne, choroby metaboliczne, choroby przewlekłe

## Abstract

*Iron is an element whose content in the human organism remains under strict control not only due to its involvement in many life processes but also because of its potential toxicity. The latest studies in iron metabolism, especially the involvement of hepcidin, which is the main regulator of iron homeostasis, broadened our knowledge in many medico/ fields (immunology, nephrology, hematology, gastrology). The present paper is a review of the literature devoted to the importance of hepcidin under selected conditions*.

## Streszczenie

*Żelazo jest pierwiastkiem, którego zawartość w organizmie podlega ścisłej regulacji ze względu na jego niezbędny udział w licznych procesach życiowych, ale również ze względu na potencjalny efekt toksyczny nadmiaru żelaza. Współczesne dane na temat gospodarki żelazem, a zwłaszcza udziału w niej głównego regulatora, jakim jest hepcydyna, wzbogaciły wiedzę w wielu dziedzinach medycyny (immunologia, nefrologia, hematologia, gastrologia). Praca stanowi przegląd piśmiennictwa dotyczącego znaczenia hepcydyny w wybranych stanach chorobowych*.

Only a few years ago the only tests for evaluating iron metabolism were blood iron, transferrin and ferritin levels and the iron binding capacity. Since 2001 iron homeostasis investigations have been expanded by new markers: hepcidin, ferroportin, hemojuvelin, erythroferrone. The genetic bases of iron metabolism have been determined *(HFE, TFR2, HJV, HAMP, SLC40A1* genes). Those discoveries opened the way for new diagnostic and therapeutic opportunities. Hence, studies on iron metabolism have become the focus of interest for scientists and doctors specializing in many fields.

Recent discoveries in the field of iron metabolism and the mechanisms of iron absorption and use demonstrated that hepcidin was the pivoting element of iron homeostasis [1]. Hepcidin is a short peptide (a chain of 25 amino acids) produced mainly in the liver. Its purpose is to release iron from macrophages and red blood cells (RBCs) into the blood. Hepcidin is also an acute phase protein. In the course of the transport of iron from the above-mentioned cells into the blood, hepcidin acts on ferroportin – a protein located in the cellular membrane of enterocytes, macrophages, and hepatocytes, and acting as a receptor for hepcidin. After combining with the receptor, hepcidin inactivates ferroportin and inhibits iron transport into the extracellular space. This leads to sideropenia, followed by anaemia. Increased hepcidin content is a result of increased iron storage, inflammations, and the activity of erythropoiesis. The opposite situation occurs under conditions associated with decreased hepcidin level, e.g. in the course of increased erythropoiesis, and in hypoxia. Besides the level of hepcidin, other factors regulating the activity of ferroportin are iron regulatory proteins (IRP1 and IRP2) [2]. Hepcidin and ferroportin are the last effectors of molecular reaction pathways participating in the complex mechanisms of iron metabolism. A discussion of the subsequent stages leading to the regulation of hepcidin expression is beyond the scope of this paper. The aim of this review is to present some current data regarding hepcidin and iron metabolism in selected diseases on the basis of a review of the literature.

The association between the susceptibility to infections and the organisms iron resources is a subject of numerous studies. It is particularly important in those populations where the rate of iron deficient individuals reaches several dozen percent, and the danger of mass epidemic outbreaks (malaria, HIV infection, parasitic diseases) is high [3]. The World Health Organisation monitors scientific reports regarding the possibility of iron supplementation in preventing infections, but as for now, the results of scientific research do not make it possible to formulate any general guidelines. On the one hand it is known that iron deficiency has a negative effect on the cellular response of the immunological system. On the other, pathogenic microorganisms use iron for their own metabolism [4]. In 2006, an extensive study of the population in Tanzania demonstrated that iron supplementation was associated with increased malaria morbidity and mortality [5]. Wegmüller et al. [6] studied children with anaemia, assessing the level of hepcidin, and trying to use the results obtained to determine those groups of patients who would significantly benefit from iron supplementation and not simultaneously increase their risk of infection. On an animal model, Agoro et al. [7] demonstrated that moderate iron supplementation in mice increased the level of hepcidin, reduced the levels of pro-inflammatory cytokines and increased response of T cells to infection with bovine tubercle bacillus.

The level of hepcidin as an acute phase protein is increased in the course of pneumonia. The increase leads to the so-called anaemia of inflammation through the inhibition of erythropoiesis (reduced iron availability as a result of inhibiting absorption and liberation of the element). On the other hand, anaemia and tissue hypoxia activates erythropoiesis by the inhibition of hepcidin. The transient anaemia accompanying pneumonia seems to have a beneficial effect on the limitation of bacterial development. Michels et al. [8] demonstrated an increased hepcidin level in the course of bacterial pneumonia in mice. The authors concluded that the associated decreased iron level reduced the risk of disseminating pathogenic bacteria in the blood.

Respiratory infections are an important health problem in the paediatric population. Some reports regarding studies in disorders of iron metabolism in the course of infections of the respiratory system, particularly the lower respiratory tract, are available in the literature [9]. The low number of those reports and some interesting conclusions that may be drawn from them constitute a foundation for continuing research in that field. Determination of hepcidin levels in pneumonia patients provided an explanation of the drop in the haemoglobin level commonly encountered in various infections [10], e.g. those caused by pneumococci. Pro-inflammatory cytokines, such as IL6, secreted in response to an infection, stimulate the synthesis of hepcidin. Hepcidin combines with ferroportin on cellular membrane of macrophages and blocks the liberation of iron, thus limiting its availability to erythropoiesis [11].

There are reports of attempts to use the assessment of iron metabolism parameters in the course of severe systemic infections in order to predict the course of the disease. It was observed that there is an unfavourable prognosis regarding survival in patients with sepsis whose blood iron levels were high. A possible application of iron chelating drugs was suggested in the therapy of critically ill patients [12]. Houamel et al. demonstrated on an animal model that switching off the hepcidin expression by means of a genetic modification leads to a higher intensity of urinary tract infections caused by *Escherichia coli* compared to individuals reacting to bacterial infection with increased expression of hepcidin. The authors also demonstrated that bacteria were able to restrict the synthesis of hepcidin in the renal cells of infected animals. That was their protection mechanism [13].

There are numerous reports regarding iron metabolism in chronic inflammations of various aetiology [14]. The authors indicate the use of hepcidin concentration assays in the diagnosis of anaemia accompanying chronic inflammation in order to distinguish it from deficient anaemia [15]. In anaemia occurring in inflammations, hepcidin levels were significantly higher. It is possible to implement appropriate treatment by distinguishing these two conditions. In patients with rheumatoid arthritis, the prohepcidin level was positively correlated with inflammatory parameters and autoimmune markers of the disease [16]. Non-specific inflammatory bowel diseases (IBD) are also a group of chronic diseases of inflammatory aetiology which are a subject of research on iron metabolism [1]. Anaemia is a common health problem in the group of patients with IBD, causing some diagnostic and therapeutic difficulties. The aetiology of anemia in this group of patients is complex. The discovery of hepcidin, proteins regulating its expression, receptors for compounds involved in signal transduction for the activation of these proteins, made it possible to differentiate between deficient, inflammatory, or drug-induced anaemia in IBD. However, study results are not unequivocal, and laboratory methods remain beyond the scope of potential commercial use. Krawiec et al. [17] assessed iron metabolism in patients with non-specific inflammatory bowel diseases and found significantly lower hepcidin levels in this group compared to the control. Lower hepcidin levels were observed particularly in patients with iron deficiency anaemia. According to the authors, that fact confirms a significant effect of the iron level on the expression of hepcidin.

One of the inflammatory disorders for which the use of iron parameters for prognostic purposes is considered is Kawasaki disease. Particularly patients with a prolonged course of this disease are at a risk of anaemia. The hepcidin level was significantly higher in patients demonstrating resistance to immunoglobulin therapy, who were at a higher risk of developing lesions in coronary arteries [18] . The authors also discuss the important problem of using high doses of acetylsalicylic acid in patients with Kawasaki disease, questioning the justifiability of such treatment. High doses of acetylsalicylic acid delay the reduction of inflammatory factors, including hepcidin, thus accounting for anaemia. However, further research is necessary to formulate therapeutic recommendations.

The role of chronic inflammation associated with the synthesis of cytokines, chemokines and growth factors secreted by adipose tissue has been underlined in the pathogenesis of obesity for several years. Problems of iron metabolism in obese people, in the context of the variability of hepcidin concentration, are of interest due to the tendency to anaemia and an inferior efficacy of treatment with iron oral preparations in obese children [19] . Moreno-Navarrete et al. [20] demonstrated that hepcidin level was positively correlated with BMI, and that dietetic intervention decreased the synthesis of hepcidin, thus improving the parameters of iron metabolism.

Type 2 diabetes is currently regarded as a disease associated with iron overload. Increased hepcidin levels contribute to the development of insulin resistance and type 2 diabetes [21]. However, the mechanism of that correlation remains unclear and needs to be studied. What is being considered are the possible effect of insulin on the regulation of hepcidin expression, the effect of glucose in the liberation of hepcidin from the pancreas, and the increased susceptibility of peripheral glucose receptors to increased hepcidin and ferritin levels [22].

Discoveries of factors involved in iron metabolism, including hepcidin, opened the way to progress in developing knowledge about the diseases whose aetiology is primarily associated with the disruption of this metabolism. Patients with β-thalassemia of pathogenetic origin, and also patients requiring frequent blood transfusions for therapeutic reasons, demonstrate an excessive tissue storage of iron from decomposed RBCs. Increased iron absorption from the alimentary system is observed in such cases. In this group, hepcidin levels are lower compared to healthy individuals [23]. Similar observations were made for patients with congenital hemochromatosis. The condition results from genetic mutations of factors responsible for the regulation of hepcidin expression [24]. Increased hepcidin level would constitute optimal treatment preventing iron accumulation in the tissues of hemochromatosis patients.

The liver is the principal place of hepcidin synthesis. Dysfunction of that organ in the course of chronic conditions, regardless the aetiology, leads to disturbed regulation of expression and subsequent reduced production of hepcidin [25]. That, in turn, accounts for the storage of iron in the liver and development of complications, such as hepatic fibrosis, and even to an increased risk of hepatic cancer. It was demonstrated that the hepcidin to ferritin concentration ratio may be used as a negative prognostic parameter in advanced liver disease in children [26]. In severe fibrosis of the liver, the hepcidin to ferritin ratio is lower, compared to that observed in children with less intensive fibrotic lesions. The study of alcoholic liver damage demonstrated the role of alcohol as a factor participating in inhibiting the expression of the hepcidin gene [27].

Kidneys are organs playing a significant role in iron metabolism. Plasma hepcidin is eliminated mostly through the kidneys – the compound is almost totally filtered in the renal glomeruli, but then re-uptaken and disrupted in the proximal tubules. As a result, only a low percent of hepcidin is eliminated in a non-altered form [28]. In patients with impaired filtration function of the kidneys, hepcidin levels are increased, which results in the lower availability of iron for erythropoiesis and intensified anaemia that is inseparably associated with chronic renal disease caused by erythropoietin deficiency [29, 30]. The use of hepcidin as a marker of iron resources in the body of patients with chronic kidney disease is a subject of ongoing research. Using such a marker would facilitate the treatment of anaemia in this group of patients. Besides participation in the pathogenesis of anaemia, hepcidin also plays a role in the pathogenesis of kidney damage via iron-dependent mediators of oxidative stress.

The results of scientific research on the complex process of iron metabolism constitute the basis for pharmacological tests of their practical application. Attempts have been made to synthesize drugs that may be used in therapy by influencing iron metabolism. Studies on antagonists and agonists of hepcidin are underway. One of them is lexaptepid [31], an antagonist of hepcidin, that may prove effective in the treatment of anaemia accompanying acute inflammations. Attempts have been made to use an antibody against hepcidin in non-specific inflammatory bowel disease [1]. Research on the application of modified heparin (possessing no anti-coagulation effect) as a hepcidin inhibitor, or antibodies against hemojuvelin – a regulator of hepcidin expression – is underway [32]. On the other hand, in the future, hepcidin agonists may be used for the treatment of thalassemia or hemochromatosis. They may be able to prevent the accumulation of iron in the tissues and associated complications [23]. Therapy with minihepcidins (synthetic polypeptides – analogies of hepcidin) and with exogenous transferrin is endeavoured. Moreover, attempts are made to perform genetic modifications of genes responsible for the coding of factors responsible for the regulation of hepcidin expression (studies on animal models) [33].

## Summary

Iron plays a principal role in the physiological function of the human organism. It has been known for a long time that its disturbed metabolism leads to such diseases as β-thalassemia and hemochromatosis. The current state of knowledge indicates that the complex mechanism of iron metabolism, with hepcidin playing the key role, is significantly correlated with the development of anaemia in the course of many diseases (inflammation-associated anaemia or anaemia of chronic diseases). The present review of the literature shows that not only is the pathogenesis of these disorders more fully understood, but some possibilities of targeted therapy are also emerging.

## References

[1] Krawiec P, Pac-Kożuchowska E (2014). Rola hepcydyny w metabolizmie żelaza w przebiegu nieswoistych zapaleń jelit. Postępy Hig Med. Dośw.

[2] Ganz T (2008). Iron homeostasis: Fitting the puzzle pieces together. Cell Metab.

[3] Jonker FAM, Te Poel E, Bates I, Boele van Hensbroek M (2017). Anaemia, iron deficiency and susceptibility to infection in children in sub-Saharan Africa, guideline dilemmas. Br J Haematol.

[4] de Pontual L (2017). [Iron and susceptibility to infections]. [Article in French]. Arch Pediatr.

[5] Sazawal S, Black RE, Ramsan M, Chwaya HM, Stoltzfus RJ, Dutta A, Dhingra U, Kabole I, Deb S, Othman MK, Kabole FM (2006). Effects of routine prophylactic supplementation with iron and folic acid on admission to hospital and mortality in preschool children in a high malaria transmission setting: Community-based, randomised, placebo-controlled trial. Lancet.

[6] Wegmüller R, Bah A, Kendall L, Goheen MM, Mulwa S, Cerami C, Moretti D, Prentice AM (2016). Efficacy and safety of hepcidin-based screen-and-treat approaches using two different doses versus a standard universal approach of iron supplementation in young children in rural Gambia: A double-blind randomised controlled trial. BMC Pediatr.

[7] Agoro R, Benmerzoug S, Rose S, Bouyer M, Gozzelino R, Garcia I, Ryffel B, Quesniaux VFJ, Mura C (2017). An Iron-Rich Diet Decreases the Mycobacterial Burden and Correlates with Hepcidin Upregulation, Lower Levels of Proinflammatory Mediators, and Increased T-Cell Recruitment in a Model of Mycobacterium bovis Bacille Calmette-Guerin Infection. J Infect Dis.

[8] Michels KR, Zhang Z, Bettina AM, Cagnina RE, Stefanova D, Burdick MD, Vaulont S, Nemeth E, Ganz T, Mehrad B (2017). Hepcidin-mediated iron sequestration protects against bacterial dissemination during pneumonia. JCI Insight.

[9] Budnevsky AV, Esaulenko IE, Ovsyannikov ES, Labzhaniya NB, Voronina EV, Chernov AV (2016). [Anemic syndrome in patients with community-acquired pneumonia]. [Article in Russian]. Klin Med (Mosk).

[10] Schoorl M, Snijders D, Schoorl M, Boersma WG, Bartels PC (2013). Transient impairment of reticulocyte hemoglobin content and hepcidin-25 induction in patients with community-acquired pneumonia. Scand J Clin Lab Invest.

[11] Wang CY, Babitt JL (2016). Hepcidin regulation in the anemia of inflammation. Curr Opin Hematol.

[12] Tacke F, Nuraldeen R, Koch A, Strathmann K, Hutschenreuter G, Trautwein C, Strnad P (2016). Iron Parameters Determine the Prognosis of Critically Ill Patients. Crit Care Med.

[13] Houamel D, Ducrot N, Lefebvre T, Daher R, Moulouel B, Sari MA, Letteron P, Lyoumi S, Millot S, Tourret J, Bouvet O, Vaulont S, Vandewalle A, Denamur E, Puy H, Beaumont C, Gouya L, Karim Z (2016). Hepcidin as a major component of renal antibacterial defenses against uropathogenic Escherichia coli. J Am Soc Nephrol.

[14] Mahajan G, Sharma S, Chandra J, Nangia A (2017). Hepcidin and iron parameters in children with anemia of chronic disease and iron deficiency anemia. Blood Res.

[15] Weiss G (2015). Anemia of chronic disorders: New diagnostic tools and new treatment strategies. Semin Hematol.

[16] Stefanova KI, Delcheva GT, Maneva AI, Batalov AZ, Geneva-Popova MG, Karalilova RV, Simitchiev KK (2016). Pathobiochemical mechanisms relating iron homeostasis to parameters of inflammatory activity and autoimmune disorders in rheumatoid arthritis. Folia Med (Plovdiv).

[17] Krawiec P, Mroczkowska-Juchkiewicz A, Pac-Kożuchowska E (2017). Serum hepcidin in children with inflammatory bowel disease. Inflamm Bowel Dis.

[18] Huang YH, Kuo HC, Dallinger R (2017). Anemia in Kawasaki disease: Hepcidin as a potential biomarker. Int J Mol Sci.

[19] Hutchinson C (2016). A review of iron studies in overweight and obese children and adolescents: a double burden in the young?. Eur J Nutr.

[20] Moreno-Navarrete JM, Moreno M, Puig J, Blasco G, Ortega F, Xifra G, Ricart W, Fernández-Real AM (2017). Hepatic iron content is independently associated with serum hepcidin levels in subjects with obesity. Clin Nutr.

[21] Andrews M, Soto N, Arredondo-Olguin M (2015). Association between ferritin and hepcidin levels and inflammatory status in patients with type 2 diabetes mellitus and obesity. Nutrition.

[22] Aregbesola A, Voutilainen S, Virtanen JK, Aregbesola A, Tuomainen TP (2015). Serum hepcidin concentrations and type 2 diabetes. World J Diabetes.

[23] Makis A, Hatzimichael E, Papassotiriou I, Voskaridou E. (2017). Clinical trials update in new treatments of β-thalassemia. Am J Hematol.

[24] Kawabata H (2017). Progress in iron metabolism research. Rinsho Ketsueki The Japanese Journal of Clinical Hematology.

[25] Lyberopoulou A, Chachami G, Gatselis NK, Kyratzopoulou E, Saitis A, Gabeta S, Eliades P, Paraskeva E, Zachou K, Koukoulis GK, Mamalaki A, Dalekos GN, Simos G (2015). Low serum hepcidin in patients with autoimmune liver diseases. PLoS One.

[26] Cakir M, Erduran E, Turkmen ES, Aliyazicioglu Y, Reis GP, Cobanoglu U, Demir S (2015). Hepcidin levels in children with chronic liver disease. Saudi J Gastroenterol.

[27] Cylwik B, Chrostek L, Szmitkowski M (2008). Wpływ alkoholu na mechanizmy regulacyjne metabolizmu żelaza. [The effect of alcohol on the regulation of iron metabolism]. [Article in Polish]. Pol Merkur Lekarski.

[28] Nemeth E, Ganz T (2009). The role of hepcidin in iron metabolism. Acta Haematol.

[29] Ueda N, Takasawa K (2017). Role of Hepcidin-25 in chronic kidney disease: Anemia and beyond. Curr Med Chem.

[30] Mercadal L, Metzger M, Haymann JP, Thervet E, Boffa JJ, Flamant M, Vrtovsnik F, Houillier P, Froissart M, Stengel B (2014). NephroTest Study Group. The relation of hepcidin to iron disorders, inflammation and hemoglobin in chronic kidney disease. PLoS One.

[31] van Eijk LT, John AS, Schwoebel F, Summo L, Vauléon S, Zöllner S, Laarakkers CM, Kox M, van der Hoeven JG, Swinkels DW, Riecke K, Pickkers P (2014). Effect of the antihepcidin Spiegelmer lexaptepid on inflammation-induced decrease in serum iron in humans. Blood.

[32] Reichert CO, da Cunha J, Levy D, Maselli LMF, Bydlowski SP, Spada C (2017). Hepcidin: Homeostasis and diseases related to iron metabolism. Acta Haematol.

[33] Liu J, Sun B, Yin H, Liu S (2016). Hepcidin: A promising therapeutic target for iron disorders: A systematic review. Medicine (Baltimore).

